# Volatile compounds—the language of all kingdoms?

**DOI:** 10.1093/jxb/erab528

**Published:** 2022-01-13

**Authors:** A Corina Vlot, Maaria Rosenkranz

**Affiliations:** Helmholtz Zentrum Muenchen, Institute of Biochemical Plant Pathology, Ingolstaedter Landstr. 1, D-85764 Neuherberg, Germany; Helmholtz Zentrum Muenchen, Institute of Biochemical Plant Pathology, Research Unit Environmental Simulation, Ingolstaedter Landstr. 1, D-85764 Neuherberg, Germany

**Keywords:** Induced systemic resistance, interspecific interactions, microbial VOCs, phytohormones, plant-plant signaling, rhizosphere interactions, small volatile compounds, volatile organic compounds (VOCs)

**Volatile organic compounds (VOCs) were originally identified as communication compounds between plants and insects. Today, we know that VOCs are released by organisms of all kingdoms, including bacteria and fungi, and mediate diverse intra- and interspecific interactions both above- and below-ground. Following recent trends in this research field, the majority of reviews and research papers in this special issue focus on possible biological and ecological functions and various other aspects of microbial VOCs (mVOCs). Additional reviews and research papers highlight connections between microbe-induced plant VOCs, and their possible application in future sustainable crop protection strategies.**

Biogenic VOCs were detected as herbivore-induced plant signaling cues for the first time in the early 1980s (Baldwin and Schultz, 1983). The discovery by Dicke and Sabelis (1988) of VOC-mediated tritrophic interactions between plants, herbivores, and herbivore enemies started a new field of research that has been growing in many different directions ever since. Now, almost 40 years after the first findings on VOCs’ biological and ecological functions in plants, we know how diverse and complex VOC-mediated interspecific interactions can be. In this issue, one example of this complexity is the study of Davidovich-Rikanati *et al.* (2022) on *Pistacia* trees (*Pistacia palaestina*) and the gall-forming aphid *Baizongia pistaciae.* The authors identified that the aphid is able to reprogram the terpene pathways of the host tree, leading to the characteristic, aphid-protecting high terpene emission from the galls. This is just one example of the diverse functions of VOCs in plant–insect interactions, as reviewed in Zhou and Jander (2022). In recent years, research efforts uncovered an enormous, still largely unexplored, source of additional volatile compounds in the emissions of bacteria and fungi. The high diversity of mVOCs suggests that these compounds play important roles in microbial ecology, both above- and below-ground. Also, in addition to mediating plant–insect interactions, plant VOCs are increasingly shown to function as signaling cues within and between plants as well as between plants, microbes, and even vertebrates (Amo *et al.*, 2013; Brosset and Blande, 2022; Sharifi *et al.*, 2022).

## Microbial volatile compounds in interspecific interactions

The various functions of mVOCs as cues mediating microbe–microbe and microbe–plant interactions are highlighted by several contributions in this special issue. Whereas much is known about the chemical nature of plant-derived VOCs, or of those derived from plants and plant organs in interaction with their microbiomes, our knowledge of mVOCs has been significantly improving only recently. In this special issue, Sharifi *et al.* (2022) review the enormous potential of mVOCs to strengthen plant fitness and growth. Furthermore, Song *et al.* (2022) report the identification of the mVOCs pyrazine and 2,5-dimethylpyrazine as modulators of plant growth in the emissions of *Bacillus amyloliquefaciens.* These mVOCs inhibit growth of *Arabidopsis thaliana* plants, potentially as a result of the growth–defense trade-off following activation of salicylic acid (SA)-associated immunity in the same plants (Box 1). While the underlying molecular mechanism remains to be elucidated, Pescador *et al.* (2022) set out to fill a similar gap in our understanding of induced systemic resistance (ISR; Box 1). The authors observed significant inductions of nitric oxide (NO) in the roots of *A. thaliana* after their exposure to mVOCs derived from root beneficial fungi of the genus *Trichoderma.* Intriguingly, *Trichoderma* mVOC-induced ISR and priming of both SA- and jasmonic acid (JA)-associated defense gene expression in above-ground plant tissues depended on root NO accumulation. This is one of the first demonstrations of a molecular mechanism underlying ISR. Whereas the VOC or the VOC mixture behind the altered resistance remains unknown, previous studies have shown that *Trichoderma* spp. release a high diversity of various VOCs, among others various terpenoids and typical fungal eight-carbon (C8) VOCs (Guo *et al.*, 2021). The C8 compounds, such as 1-octen-3-ol, are probably the most studied fungal VOCs so far and can cause the typical aroma of some mushroom fruiting bodies (Pennerman *et al.*, 2022). Various fungal C8 VOCs were shown to affect the performance of other organisms, for example by reducing their growth (Pennerman *et al.*, 2022). How the functions of fungal C8 compounds differ from those of other prominent VOC groups, such as terpenes, is not yet elucidated. Fungal terpenoids in particular were recently connected to the ecological functions of fungi in an *in vitro* study (Guo *et al.*, 2021).

**Box 1. Induced systemic resistance**

Interactions between plant roots and beneficial microorganisms in the rhizosphere can prime defense responses in above-ground plant parts in a process termed induced systemic resistance (ISR; Pieterse *et al.*, 2014; Vlot *et al.*, 2021; Sharifi *et al.*, 2022). Whereas the underlying molecular mechanisms remain largely unclear, above-ground defense priming increasingly appears associated with both SA and JA defense pathways (Vlot *et al.*, 2021). One particularly well-characterized example of ISR is induced by plant growth-promoting rhizobacteria, *Pseudomonas simiae*, in *Arabidopsis thaliana* (Pieterse *et al.*, 2021). Similar to *Trichoderma-*induced ISR reported on in this issue by Pescador *et al.* (2022), the ISR response can be induced by the volatile emissions of *P. simiae* without physical contact between *A. thaliana* roots and bacteria (Martínez-Medina *et al.*, 2013, 2017; Zamioudis *et al.*, 2015). ISR induced by *P. simiae* or *Trichoderma* spp. VOCs depends on the *A. thaliana* root-expressed transcription factor MYB72 (Van der Ent *et al.*, 2008; Segarra *et al.*, 2009). In this Special Issue, this MYB72-dependent ISR mechanism in response to *Trichoderma* VOCs is shown to additionally depend on root NO accumulation acting upstream of MYB72 (Pescador *et al.*, 2022). Together, mVOCs, NO, and MYB72 might be general signaling components of ISR. In this issue, Song *et al.* (2022) report data which add yet another, intriguing, level of complexity to the interaction of plants with ISR-inducing plant growth-promoting bacteria. In their study VOCs of *Bacillus amyloliquefaciens* both promote and inhibit *A. thaliana* growth depending on VOC intensity/concentration at the root surface. Future research is required to further unravel the molecular mechanisms downstream of ISR-inducing mVOCs and the complex, in part antagonistic, responses that are triggered by different VOC concentrations and probably also composition, especially if we also take into account further possible ‘interference’ by microbiome-derived mVOCs.

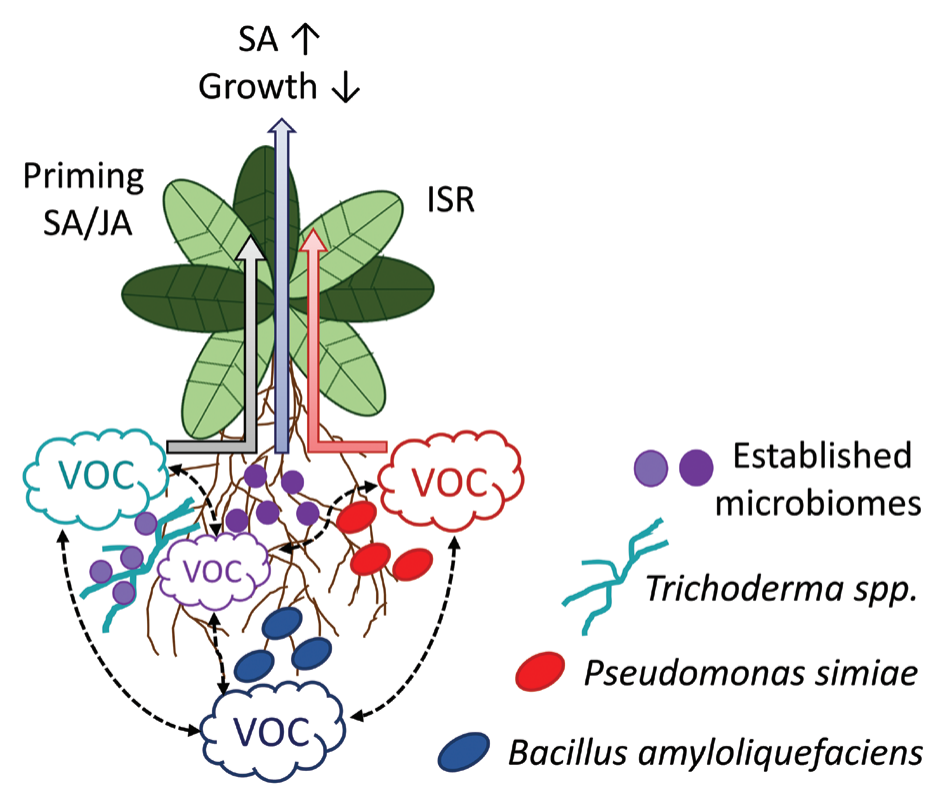

Microbes release not only VOCs but also more simple, inorganic volatiles (Das *et al.*, 2022; Gámez-Arcas *et al.*, 2022). Such small volatile compounds with a molecular weight <45Da can also have ecological functions mediating interactions between plants, bacteria, and fungi. Gámez-Arcas *et al.* (2022) review current evidence suggesting that compounds such as NO and CO can signal to the plant about the presence of microbes. It is intriguing that the receiver plant can enhance its photosynthesis and growth upon perceiving these compounds. It remains, however, to be explored which roles the small microbial volatiles play in distinct interspecific interactions compared with more complex VOCs. The functions of small bacterial volatiles seem, however, not to be restricted to promoting plant growth. They may also be involved in improving plant performance indirectly: Das *et al.* (2022) show that plant growth-promoting bacterial volatiles can have antagonistic properties against phytopathogens. This is illustrated by new findings revealing the effects of *Serratia plymuthica* volatiles, including ammonia, on membrane integrity of pathogenic fungi. In this interaction, bacterial volatile compounds might thus influence fungistasis in the rhizosphere and by this means potentially in turn also plant homeostasis.

## Plant VOCs and sustainable crop protection

Accumulating evidence points to a prominent role for plant VOCs in plant–plant propagation of, for example, defense reactions. When we consider VOC detection in biological systems, insects are known to perceive odors in their peripheral nervous system (Zhou and Jander, 2022). It remains, however, not well understood how plants perceive and respond to distinct VOCs. Nevertheless, studies have revealed altered plant defense upon exposure to mVOCs or to the VOC emissions of neighboring plants (Zamioudis *et al.*, 2015; Martínez-Medina *et al.*, 2017; Riedlmeier *et al.*, 2017; Wenig *et al.*, 2019; Frank *et al.*, 2021). In this special issue, current knowledge on plant–plant signaling is reviewed by Brosset and Blande (2022): the authors list altogether 40 different plant-derived VOCs that were proven to alter receiver plant resistance or defense-related internal signaling. Thus, it seems likely that detection of various VOCs and the subsequent adjustments in plant internal signaling rely on more than one or more specific receptors in plants. Brosset and Blande (2022) discuss current data, which support a different mechanism of VOC recognition in receiver plants, amongst others relying on uptake and subsequent conversion of VOCs to soluble metabolites. Metabolizing VOCs may be an effective functional mechanism of plant–plant signaling as it could allow compound-specific responses in a dose-dependent manner. How the different volatile cues are integrated into a plant response remains to be elucidated.

In addition to acting as signaling intermediates in different biological processes, VOCs are well known for their more ‘direct’ repellant and attractant properties, which have already found use in sustainable agriculture. A prominent example is the use of VOCs in so-called push–pull systems (Pickett and Khan, 2016; Brilli *et al.*, 2019; Zhou and Jander, 2022). Here, crop plants are grown in intercropping systems, where the VOC pattern of one intercropped plant species deters herbivores, while another species, usually planted on the side of the field, attracts the pest away from the main crop (Pickett and Khan, 2016; Zhou and Jander, 2022). Similar to push–pull intercropping in plant protection from insects, plant–plant or microbe–plant communication could be applied to improve plant performance, for example by priming defense against pathogens (Brilli *et al.*, 2019; Vlot *et al.*, 2021). Lazazzara *et al.* (2022) suggest in this issue, that viticulture in particular could become more sustainable by applying VOC-based plant protection. Intercropping grapevine with a species that on the one hand can physically support grapevine growth and on the other hand chemically primes the wine defense system against common pathogens could be especially profitable. Similarly, experimental evidence of plant to plant propagation of systemic acquired resistance (SAR) signaling in the cereal crop barley is provided by Brambilla *et al.* (2022). They suggest that VOC-based sustainable crop protection strategies could also be beneficial for cereal crops, and report that SAR-associated volatile emissions of barley include nonanal and the terpenoid β-ionone.

## Future directions

The last 40 years of VOC research have been fascinating, but the coming decades do not seem likely to become less exciting. Combining the information from the articles in this special issue, it becomes evident that intra- and interspecific interactions are driven to a large extent by volatile compounds, and can influence the fitness of both microbes and plants. At the same time, many intriguing questions remain. For example, the evolutionary advantages of the distinct, species-specific VOC patterns remain unclear. In future, the roles of VOCs and full VOC bouquets should be deciphered as near to natural environments as possible (Weisskopf *et al.*, 2021). Indeed, whereas increasingly more is known about the chemical nature of plant and microbial VOCs, experimental restrictions continue to obscure the dynamics and associated diversity of these compounds, especially in the rhizosphere (Sharifi *et al.*, 2022). In future, new experimental designs, such as those suggested by Sharifi *et al.* (2022), should allow natural gas exchange and the movement of compounds in the free atmosphere or, for below-ground interactions, in soil-like structures. The data from such approaches should provide insights into VOC dynamics in multi-organism interactions, for example plants interacting with fungi, which each interact with their own microbiome (Box 1). Such approaches can be coupled with simpler laboratory studies, allowing the uncovering of causal links in the functions of VOCs (as suggested by Das *et al.*, 2022). Less attention has so far also been paid to possible connections between plant above-ground and below-ground interactions, including those occurring during ISR (Box 1). Integrated approaches and interdisciplinarity can aid in filling current knowledge gaps and shed light on the principles and causalities that drive VOC-mediated intra- and interspecific interactions in different natural ecosystems.

## References

[CIT0001] Amo L, Jansen JJ, van Dam NM, Dicke M, Visser ME. 2013. Birds exploit herbivore-induced plant volatiles to locate herbivorous prey. Ecology Letters 16, 1348–55. 24103093 10.1111/ele.12177

[CIT0002] Baldwin IT, Schultz JC. 1983. Rapid changes in tree leaf chemistry induced by damage: evidence for communication between plants. Science 221, 277–9. 17815197 10.1126/science.221.4607.277

[CIT0003] Brambilla A, Sommer A, Ghirardo A, Knappe C, Wenig M, Weber B, Amesmaier M, Lenk M, Schnitzler JP, Vlot AC. 2022. Immunity-associated volatile emissions of β-ionone and nonanal propagate defence responses in neighbouring barley plants. Journal of Experimental Botany 73, 615–630. 10.1093/jxb/erab520 34849759

[CIT0004] Brilli F, Loreto F, Baccelli I. 2019. Exploiting plant volatile organic compounds (VOCs) in agriculture to improve sustainable defense strategies and productivity of crops. Frontiers in Plant Science 10, 264. 30941152 10.3389/fpls.2019.00264 PMC6434774

[CIT0005] Brosset A, Blande JD. 2022. Volatile-mediated plant–plant interactions: volatile organic compounds as modulators of receiver plant defence, growth, and reproduction. Journal of Experimental Botany 73, 511–528. 10.1093/jxb/erab487 PMC8757495 34791168

[CIT0006] Das P, Effmert U, Baermann G, Quella M, Piechulla B. 2022. Impact of bacterial volatiles on phytopathogenic fungi: an in vitro study on microbial competition and interaction. Journal of Experimental Botany 73, 596–614. 10.1093/jxb/erab476 34718549

[CIT0007] Davidovich-Rikanati R, Bar E, Hivert G, et al. 2022. Transcriptional up-regulation of host-specific terpene metabolism in aphid-induced galls of *Pistacia palaestina*. Journal of Experimental Botany 73, 555–570. 10.1093/jxb/erab289 34129033

[CIT0008] Dicke M, Sabelis MW. 1988. How plants obtain predatory mites as bodyguards. Netherlands Journal of Zoology 38, 148–165.

[CIT0009] Frank L, Wenig M, Ghirardo A, van der Krol A, Vlot AC, Schnitzler JP, Rosenkranz M. 2021. Isoprene and β-caryophyllene confer plant resistance via different plant internal signaling pathways. Plant, Cell & Environment 44, 1151–1164. 10.1111/pce.14010 33522606

[CIT0010] Gámez-Arcas S, Baroja-Fernández E, Garcia-Gomez P, Munoz FJ, Almagro G, Bahaji A, Sanchez-Lopez AM, Pozueta-Romero J. 2022. Action mechanisms of small microbial volatile compounds in plants. Journal of Experimental Botany 73, 498–510. 10.1093/jxb/erab463 34687197

[CIT0011] Guo Y, Jud W, Weikl F, Ghirardo A, Junker RR, Polle A, Benz JP, Pritsch K, Schnitzler JP, Rosenkranz M. 2021. Volatile organic compound patterns predict fungal trophic mode and lifestyle. Communications Biology 4, 673. 34083721 10.1038/s42003-021-02198-8 PMC8175423

[CIT0012] Lazazzara V, Avesani S, Robatscher P, Oberhuber M, Pertot I, Schuhmacher R, Perazzolli M. 2022. Biogenic volatile organic compounds in the grapevine response to pathogens, beneficial microorganisms, resistance inducers, and abiotic factors. Journal of Experimental Botany 73, 529–554. 10.1093/jxb/erab367 34409450

[CIT0013] Martínez-Medina A, Fernández I, Sánchez-Guzmán MJ, Jung SC, Pascual JA, Pozo MJ. 2013. Deciphering the hormonal signaling network behind the systemic resistance induced by *Trichoderma harzianum* in tomato. Frontiers in Plant Science 4, 206. 23805146 10.3389/fpls.2013.00206 PMC3690380

[CIT0014] Martínez-Medina A, Van Wees SCM, Pieterse CMJ. 2017. Airborne signals by *Trichoderma* fungi stimulate iron uptake responses in roots resulting in priming of jasmonic acid- dependent defences in shoots of *Arabidopsis thaliana* and *Solanum lycopersicum*. Plant, Cell & Environment 40, 2691–2705. 10.1111/pce.13016 28667819

[CIT0015] Pennerman KK, Yin G, Bennett JW. 2022. Eight-carbon volatiles: prominent fungal and plant interaction compounds. Journal of Experimental Botany 73, 487–497. 10.1093/jxb/erab438 34727164

[CIT0016] Pescador L, Fernandez I, Pozo MJ, Romero-Puertas MC, Pieterse CMJ, Martinez-Medina A. 2022. Nitric oxide signalling in the root is required for MYB72-dependent systemic resistance induced by *Trichoderma* volatiles in Arabidopsis. Journal of Experimental Botany 73, 584–595. 10.1093/jxb/erab294 PMC8757496 34131708

[CIT0017] Pickett JA, Khan ZR. 2016. Plant volatile-mediated signalling and its application in agriculture: successes and challenges. New Phytologist 212, 856–870. 10.1111/nph.14274 27874990

[CIT0018] Pieterse CMJ, Berendsen RL, de Jonge R, et al. 2021. *Pseudomonas simiae* WCS417: star track of a model beneficial rhizobacterium. Plant and Soil 461, 245–263.

[CIT0019] Pieterse CMJ, Zamioudis C, Berendsen RL, Weller DM, Van Wees SCM, Bakker PAHM. 2014. Induced systemic resistance by beneficial microbes. Annual Review of Phytopathology 52, 347–375. 10.1146/annurev-phyto-082712-102340 24906124

[CIT0020] Riedlmeier M, Ghirardo A, Wenig, M, Knappe C, Koch K, Georgii E, Dey S, Parker JE, Schnitzler JP, Vlot AC. 2017. Monoterpenes support systemic acquired resistance within and between plants. The Plant Cell 29, 1440–1459. 28536145 10.1105/tpc.16.00898 PMC5502447

[CIT0021] Segarra G, Van Der Ent S, Trillas I, Pieterse CMJ. 2009. MYB72, a node of convergence in induced systemic resistance triggered by a fungal and a bacterial beneficial microbe. Plant Biology 11, 90–96. 19121118 10.1111/j.1438-8677.2008.00162.x

[CIT0022] Sharifi R, Jeon JS, Ryu CM. 2022. Belowground plant–microbe communications via volatile compounds. Journal of Experimental Botany 73, 463–486. 10.1093/jxb/erab465 34727189

[CIT0023] Song CG, Jeon JS, Sim HJ, Lee S, Jung J, Kim SG, Moon SY, Ryu CM. 2022. Dual functionality of natural mixtures of bacterial volatile compounds on plant growth. Journal of Experimental Botany 73, 571–583. 10.1093/jxb/erab466 34679179

[CIT0024] Van der Ent S, Verhagen BWM, Van Doorn R, et al. 2008. MYB72 is required in early signaling steps of rhizobacteria-induced systemic resistance in Arabidopsis. Plant Physiology 146, 1293–304. 18218967 10.1104/pp.107.113829 PMC2259080

[CIT0025] Vlot AC, Sales JH, Lenk M, Bauer K, Brambilla A, Sommer A, Chen Y, Wenig M, Nayem S. 2021. Systemic propagation of immunity in plants. New Phytologist 229, 1234–1250. 10.1111/nph.16953 32978988

[CIT0026] Weisskopf L, Schulz S, Garbeva P. 2021. Microbial volatile organic compounds in intra-kingdom and inter-kingdom interactions. Nature Reviews Microbiology 19, 391–404. 33526910 10.1038/s41579-020-00508-1

[CIT0027] Wenig M, Ghirardo A, Sales JH, et al. 2019. Systemic acquired resistance networks amplify airborne defense cues. Nature Communications 10, 3813. 10.1038/s41467-019-11798-2 PMC6707303 31444353

[CIT0028] Zamioudis C, Korteland J, Van Pelt JA, et al. 2015. Rhizobacterial volatiles and photosynthesis-related signals coordinate MYB72 expression in Arabidopsis roots during onset of induced systemic resistance and iron-deficiency responses. The Plant Journal 84, 309–322. 26307542 10.1111/tpj.12995 PMC5019235

[CIT0029] Zhou S, Jander G. 2022. Molecular ecology of plant volatiles in interactions with insect herbivores. Journal of Experimental Botany 73, 449–462. 10.1093/jxb/erab413 34581787

